# Postoperative infusion of dexmedetomidine via intravenous patient-controlled analgesia for prevention of postoperative delirium in elderly patients undergoing surgery

**DOI:** 10.1007/s40520-023-02497-6

**Published:** 2023-07-20

**Authors:** Kangjie Xie, Jinyan Chen, Lili Tian, Fulei Gu, Yafei Pan, Zhangxiang Huang, Jun Fang, Weifeng Yu, Huidan Zhou

**Affiliations:** 1 Department of Anesthesiology, Zhejiang Cancer Hospital, Research Center for Neuro-Oncology Interaction, Hangzhou Institute of Medicine (HIM), Chinese Academy of Sciences, Hangzhou, 310022 Zhejiang China; 2 Postgraduate training Base Alliance of Wenzhou Medical University (Zhejiang Cancer Hospital), Hangzhou, 310022 Zhejiang China; 3 Traditional Chinese Medicine Pharmacy, Zhejiang Hospital, Hangzhou, 310022 Zhejiang China

**Keywords:** Elderly patients, Postoperative delirium, Dexmedetomidine

## Abstract

**Background:**

Postoperative delirium (POD) is a common clinical complication in elderly patients after surgery and predicts poor outcomes.

**Aim:**

We researched whether postoperative infusion of dexmedetomidine (DEX) had prophylactic effect on POD in elderly patients.

**Methods:**

A total of 236 patients over the age of 60 years undergoing thoracoabdominal tumor surgery were enrolled in Zhejiang Cancer Hospital from November 2016 to October 2020. The patients were randomly assigned into DEX group (group D) and control group (Group C). DEX was provided via PCIA pump 1–3 days after surgery, which consisted of 3 ug/kg sufentanil and 3 ug/kg DEX in group D, and 3 ug/kg sufentanil without DEX in group C. The PCIA parameters were programmed as follows: total amount 150 ml, 2 ml bolus dose with a lock-out of 10 min and background infusion rate 2 ml/h. The primary endpoint was the incidence of POD, assessed twice daily within 7 days after surgery by Richmond Agitation-Sedation Scale (RASS) and the Confusion Assessment Method–Intensive Care Unit (CAM-ICU). The secondary endpoint was postoperative hospitalization days, ICU stay time, adverse events and non-delirium complications.

**Results:**

The incidence of POD in all patients was 7%. The incidence of POD in group C was significantly higher than that in group D (10.1% vs 3.4%, *P* = 0.042). There were no significant differences in length of hospital stay after operation, ICU stay time, the percentage of patients discharged within 7 days after surgery, non-delirium complications, and 30-day all-cause deaths between the two groups. The incidence of hypertension in group D was lower than that in group C (*P* = 0.003), and there were no differences in other adverse events.

**Conclusion:**

Patients aged over 60 years received DEX in addition to intravenous patient-controlled analgesia (PCIA) for major thoracoabdominal surgery experienced less delirium.

**Supplementary Information:**

The online version contains supplementary material available at 10.1007/s40520-023-02497-6.

## Introduction

After surgery, elderly people often have symptoms such as postoperative agitation uncontrolled movement, nonsense, placement or orientation issue, hallucination, sleeplessness or lethargy, apathy, and so on. These symptoms imply that the patients may suffer from perioperative neurocognitive dysfunction (PND). However, it may have an impact on patients' life quality and poor organ functional recovery following surgery, leading to an increase in mortality [1].

Postoperative delirium (POD), one of PND, occurs during the surgical recovery period, so it complicates postoperative care and introduces new hazards, such as aggressive fluid removal and fractures caused by falls, which increase the load on hospital resources and the family. Delirium can contribute to prolonged length of stay, readmission rates, increased institutional discharge, and ultimately, high resource utilization [2].

Delirium is more common in older patients after surgery, so preventing and treating POD is a pressing clinical issue. Delirium is multicausal. Previous studies on delirium pointed out old age, cognitive impairment, medical comorbidity, institutional residence, psychotropic drug, visual and hearing impairment are most predisposing risk factors, while admission to ICU, anticholinergic drugs, alcohol or drug withdrawal, infections, iatrogenic complications, metabolic derangements and pain are most precipitating factors [3, 4]. Effective preventive measures include depth of anesthesia monitoring, intraoperative DEX infusion, and multimodal analgesia [5]. Effective drugs for treating delirium are haloperidol, olanzapine and quetiapine [6]. Sanders RD demonstrated that DEX may reduce isoflurane-induced neurocognitive impairment in developing brains. Besides, DEX, a highly selective α_2_-adrenergic agonist, produces equivalent pharmacological effects such as sedative, analgesic, anti-anxiety in the central and peripheral nervous systems by acting on distinct subtypes of α_2_-adrenergic receptors [7]. According to a meta-analysis, perioperative DEX may result in decreased incidence of POD in patients undergoing non-cardiac surgery [8]. Deiner S, on the other hand, discovered that DEX did not reduce incidence of POD [9]. As a result, whether DEX could prevent POD remains controversial. So far, no research has been done to determine if DEX delivered by Patient-Controlled Intravenous Analgesia (PCIA) can prevent POD.

Hence, the current research was planned to look into the impact of DEX given by PCIA on POD in elderly patients after major thoracoabdominal surgery. We anticipated that DEX given through PCIA 1–3 days after surgery might prevent POD in older patients.

## Materials and methods

### Study design

This prospective, randomized, controlled trial conformed to the ethical guidelines of the 1975 Declaration of Helsinki and was approved by the Ethics Committee of Zhejiang Cancer Hospital ([2015]-01–13), and the study was registered before patient enrollment at clinicaltrials.gov with registration No. NCT02923128. Written informed consent was obtained from all patients prior to the study.

### Participants

The inclusion criteria were patients aged 60 years or older who underwent elective surgery for gastrointestinal or lung tumors in Zhejiang Cancer Hospital. The exclusion criteria were: (1) patients with a clear preoperative history of nervous system and mental system diseases or long-term use of sedatives or antidepressants; (2) history of alcoholism, drug abuse or drug dependence; (3) have a history of brain surgery or injury; (4) serious visual or hearing impairment; (5) patients who failed to complete the cognitive function test or refused to participate in the study; (6) sick sinus syndrome, second-degree or greater atrioventricular block or other contraindications for use of α_2_ adrenergic agonist; (7) renal failure requiring dialysis or hepatic dysfunction.

### Protocol

Patients were treated without premedication. Throughout the perioperative period, electrocardiogram, pulse oxygen saturation, invasive arterial pressure and end-tidal concentrations of carbon dioxide monitoring were necessary. All of patients underwent the operation under total intravenous general anesthesia. Anesthesia was induced with oxycodone (0.2–0.3 mg/kg), propofol (1.0–1.5 mg/kg) and rocuronium (0.9 mg/kg). Intermittent positive pressure ventilation was used after tracheal intubation, with tidal volume of 6–8 ml/kg, respiratory frequency of 10–12 times/min, inspiratory/expiratory ratio of 1:2, PEEP 3–5 cmH_2_O. Anesthesia was then maintained by propofol (6–10 mg/kg/h) and remifentanil (0.1–0.3 μg/kg/min) to maintain the bispectral index between 45 and 55 and to ensure the main arterial pressure within 20% of the baseline. Several measures were taken to reduce the occurrence of delirium, including the thermal insulation measures, avoiding use of inhaled anesthetics, benzodiazepines and anticholinergic drugs, minimizing the occurrence of perioperative hypotension, hypoxemia and hypercapnia. PCIA pump was provided after surgery, which consisted of 3 ug/kg sufentanil and 3 ug/kg DEX (manufactured by Yangtze River Pharmaceutical Group Co, Ltd, China) in group D, and 3 ug/kg sufentanil without DEX in group C. The PCIA parameters were programmed as follows: total amount 150 ml, 2 ml bolus dose with a lock-out of 10 min and background infusion rate 2 ml/h.

Patients who developed POD were given nonpharmacological treatment. If patients could not cooperate with non-drug therapy, haloperidol was given intravenously. Mildly excited patients were given 0.25–0.5 mg, and severely excited patients were given 0.5–1.0 mg. If patients’ symptoms were not relieved, DEX 1ug/kg loading dose was pumped for 10–15 min, and then, 0.25–0.5 ug/kg.h dose was pumped to treat POD.

### Measurements

Demographic characters such as age, gender, body mass index (BMI) and education level were recorded. The preoperative education level, history of operation, preoperative albumin level, surgical type, surgical duration, anesthesia duration, intraoperative liquid volume, perioperative blood transfusion, ICU admission, the hours in the ICU and length of stay in hospital after surgery were recorded.

Delirium assessment [10]: Our primary outcome was the 7-day incidence of postoperative delirium assessed by CAM-ICU twice daily (8 a.m. and 8 p.m.) and supplemented with a review of medical and nursing records. The assessment was carried out by investigators who had been trained prior to the trial and were unaware of the group assignment. All investigators and patients were unknown of experiments and results. The four clinical criteria for CAM-ICU are as follows: (1) acute onset with fluctuating course of disease; (2) inattention; (3) altered level of consciousness; (4) disorganized thinking. Delirium can be diagnosed by the appearance of both features 1 and 2, with at least one of features 3 or 4. RASS was used to measure sedation or agitation prior to testing delirium. If the patient was deeply sedated or unable to fall asleep (RASS − 4 or − 5), delirium assessment was halted; if RASS score was of − 3 or above, delirium was evaluated by CAM-ICU. Delirium is classified into three types: hyperactive, hypoactive, and mixed subtype. Hyperactive delirium is characterized by agitation, restlessness, and attempts to remove ducts, while hypoactive delirium is characterized by apathy, lethargy, and reduced response. The term "mixed delirium" refers to a patient's features that alternate between the two. If the patients were discharged within 7 days after surgery, the POD assessment was recorded terminated and other evaluations were continued.

Pain was assessed at 8 a.m. on postoperative day 1, day 2, day 3 by NRS (Numeric Rating Scale) (The numbers 0–10 were used instead of words to indicate pain levels. Divide a straight line into 10 segments and describe pain on a scale of 0 to 10). The incidence of postoperative nausea and vomiting within 3 days after surgery was recorded.

Adverse events were recorded every day until postoperative day 3, including hypotension, hypertension, bradycardia, tachycardia, hypoxemia, and interventions (adjustment or stoup study drug infusion or intravenous administration). Hypoxemia treatment options included oxygen supplement or endotracheal intubation. Systolic blood pressure less than 90 mmHg or 25% below baseline was considered hypotension, while that more than 160 mmHg or 25% high baseline was considered hypertension. A heart rate less than 50 bpm was recorded as bradycardia, while that greater than 100 bpm was recorded as tachycardia. Oxygen saturation less than 90% was considered hypoxemia.

The occurrence of non-delirium postoperative complications as well as the 30-day all-cause mortality were recorded by follow-up.

### Statistical analysis

In our trial, through retrospective data analysis in our hospital, we found that the incidence of postoperative delirium in this group was 22%. According to a previous study [11], we hypothesized that the incidence of delirium would decrease by 3/5 in the DEX group. We estimated a sample size of 117 subjects per group would provide 80% power with α error of 0.05 by PASS 11. To allow for the possibility of missing samples, we enlarged the sample size by 3%, requiring 120 patients per group.

SPSS20.0 software was used for data entry, sorting and analysis. Measurement data of normal distribution were expressed as mean $\pm$ standard deviation and analyzed by independent-sample *t* test. The measurement data of non-normal distribution were expressed as the median (interquartile range) and analyzed by Mann–Whitney *U* test. Pearson ${x}^{2}$ test was used for comparison of count data. Statistical significance was determined at the *P* < 0.05 level.

## Results

**Fig. 1:**
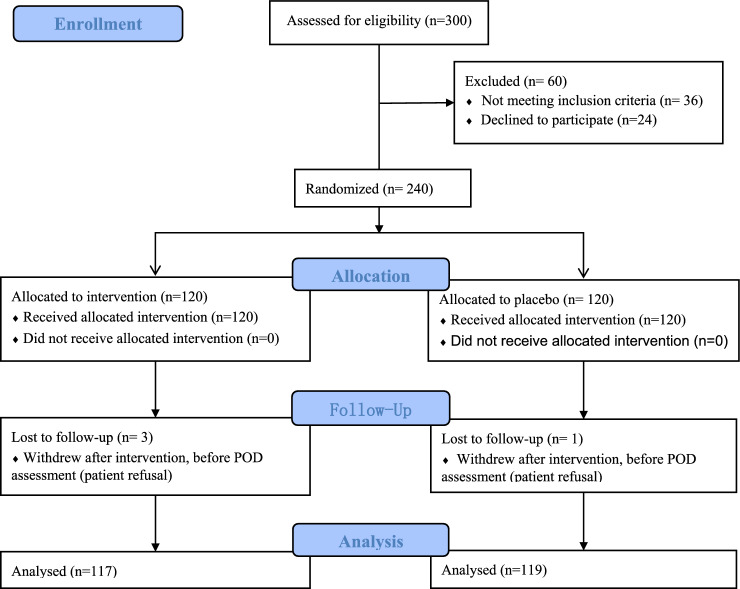
Flow diagram of patients through the study

Between November 2016 and October 2020, 300 patients were eligibility for study participation; 60 patients were excluded and 240 patients were enrolled to the study. They were randomly assigned to receive either DEX or normal saline. Four patients were excluded from the study because they did not cooperate to complete the POD assessment, and finally, 236 patients completed the study Fig. 1. There were no significant differences in sex ratio, age, BMI, educational level, operation type, history of operation, proportion of blood transfusion, ICU admission, anesthesia and operation duration, intraoperative fluid volume, preoperative and postoperative albumin level between the two groups (Table 1).

**Table 1:** Perioperative variables

Characteristics	D group (*n* = 117)	C group (*n* = 119)	*P value*
Sex (female/male)	34/83	42/77	0.305
Age (years)	67.9 ± 5.6	68.6 ± 5.5	0.821
BMI	22.3 ± 3.5	22.2 ± 3.4	0.459
Educational level, No. (%)			0.409
Illiteracy	54 (46.2)	61 (51.2)	
Primary school	36 (30.8)	36 (30.3)	
Junior high school	24 (20.5)	16 (13.4)	
High school and above	3 (2.6)	6 (5.0)	
Type of surgery, No. (%)			0.841
Abdominal (gastrointestinal)	83 (70.9)	83 (69.7)	
Thoracic (lung)	34 (29.1)	36 (30.3)	
History of surgical anesthesia, No. (%)	35(29.9)	39(32.8)	0.636
Blood transfusion, No. (%)	12 (10.3)	18 (15.1)	0.261
ICU admission, No. (%)	81 (84.8)	90 (75.6)	0.271
ICU admission with intubation	4 (3.4)	9 (7.6)	0.163
Duration of surgery (min)	173.8 ± 84.6	173.2 ± 94.4	0.964
Duration of anesthesia (min)	202.8 ± 87.6	204.0 ± 96.1	0.924
Intraoperative infusion volume (ml)	2050 ± 654	1986 ± 753	0.157
Preoperative albumin level (g/L)	40.4 ± 5.1	39.8 ± 5.2	0.378
Postoperative albumin level (g/L)	30.9 ± 4.3	30.9 ± 4.5	0.965

Date are number (%), mean (SD). BMI: body mass index, ICU: intensive care unit

In our trial, postoperative delirium occurred in 14 of 236 patients (12 in group C and 4 in group D). The incidence of POD in group C (10.1% vs 3.4%) was higher than that in group D (*P* = 0.042). The postoperative length of stay in hospital after surgery and length of stay in ICU were similar between the two groups. There was no statistically significant difference in the percentage of patients discharged within 7 days after surgery and 30-day all-cause deaths between the two groups. There was no significant difference in NRS pain scores between the two groups on 1, 2, and 3 days after surgery. The incidence of postoperative nausea and vomiting in the DEX group (3[2.6%] of 117 patients) was significantly higher than that in the control group (10[8.4%] of 119 patients, *P* = 0.049) within 3 days after operation (Table 2).

**Table 2:** Effectiveness outcomes

	D group (*n* = 117)	C group (*n* = 119)	*P value*
POD, No. (%)	4 (3.4)	12 (10.1)	0.042*
Drug therapy for delirium	2 (1.7)	9 (7.6)	0.033*
Length of stay in ICU (h)	12.2 ± 9.7	12.6 ± 8.0	0.229
Length of stay in hospital after surgery (d)	11.7 ± 9.1	11.6 ± 8.5	0.863
30-day all-cause mortality, No. (%)	1 (0.9)	2 (1.7)	1.000
Discharge within 7 days after surgery	33 (28.2)	35 (29.4)	0.838
NRS for pain			
The first day after surgery	2 (2—2)	2 (1—2)	1.000
The second day after surgery	2 (2—2)	2 (1—2)	1.000
The third day after surgery	1 (1—1)	2 (1—2)	1.000
Postoperative nausea and vomiting, No. (%)	3 (2.6)	10 (8.4)	0.049*

Data are mean ± SD, number (%), median (IQR). POD: Postoperative delirium, NRS: numerical rating scale; *p < 0.05

The incidence of bradycardia, tachycardia, hypotension and hypoxemia did not differ between the two groups, the same as the difference in the situation requiring intervention for the above adverse events. Meanwhile, the incidence of hypertension was significantly lower in the group D (*P* = 0.003), and the number of cases requiring intervention for hypertension was also lower (*P* = 0.011). Even more patients in the DEX group had experimental drugs adjusted than in the control group, but there was no significant difference (Table 3).

**Table 3:** Safety outcomes

	D group (*n* = 117)	C group (*n* = 119)	*P value*
Adverse events, No. (%)			
Tachycardia	34 (29)	29 (24.3)	0.415
Requiring intervention	9 (7.7)	12 (10.1)	0.519
Bradycardia	11 (9.4)	12 (10.1)	0.860
Requiring intervention	0	1 (0.8)	1
Hypotension	24 (20.5)	23 (19.3)	0.820
Requiring intervention	3 (2.6)	3 (2.5)	1
Hypertension	15 (12.8)	34 (28.6)	0.003*
Requiring intervention	8 (6.8)	21 (17.6)	0.011*
Hypoxemia	3 (2.6)	3 (2.5)	1
Requiring intervention	3 (2.6)	3 (2.5)	1
Adjustment of PCIA, No. (%)			0.580
None	108 (92.3)	112 (94.1)	
Stopped temporarily or permanently	9 (7.7)	7 (5.9)	

Data are number (%); *p < 0.05. PCIA: intravenous patient-controlled analgesia

The incidence of non-delirium complications was similar between the two groups, and the difference was not significant (Table 4).

**Table 4:** Non-delirium complications

Variable, No. (%)	D group (*n* = 117)	C group (*n* = 119)	*P value*
New arrhythmia	12 (10.3)	6 (5.0)	0.131
Heart failure	3 (2.6)	1 (0.8)	0.602
Pneumonia	4 (3.4)	6 (5.0)	0.536
Anastomotic fistula	3 (2.6)	4 (3.4)	1.000
Gastrointestinal bleed	1 (0.9)	2 (1.7)	1.000
Infection	2 (1.7)	8 (6.7)	0.056
Incision dehiscence	2 (1.7)	0 (0.0)	0.470
All	27 (23.1)	27 (22.7)	0.943

Data are presented as number (%)

## Discussion

The aim of our trial was to see whether postoperative DEX given by PCIA can reduce the development of postoperative delirium in elderly patients. Data from 236 patients were analyzed, and it was found that 3.4% (4 of 117) of group D and 10.1% (12 of 119) of group C had POD, which is statistically significant. DEX given by postoperative intravenous patient-controlled analgesia may thereby lower the incidence of POD in elderly patients. DEX can significantly reduce the incidence of postoperative nausea and vomiting without increasing adverse events, but it cannot reduce the incidence of no-delirium complications or shorten the length of stay.

Pervious [12, 13] studies showed that the incidence of POD in patients after surgery varied from 11 to 51%, with the prevalence increasing with age. Because delirium is preventable and risk factors were treated with interventions such as adequate postoperative analgesia, appropriate depth of anesthesia, and so on [5], the incidence of delirium in the placebo group was 10.1%, which was lower than previously reported. Consistent with Burkhart CS's findings, which suggested longer duration of mechanical ventilation were associated with postoperative delirium in the elderly after cardiac surgery, we found that the occurrence of POD on ICU admission was significantly higher in patients with intubation status than in patients without intubation (46.2% vs 4.7%, *p* = 0.000) [14]. Perhaps the difference was due to the fact that intubated patients with poor cardiopulmonary function were more likely to develop POD. Furthermore, we allowed these patients to tolerate tracheal catheters with sedatives, which might increase the risk of delirium [15]. However, there was no statistically significant difference in the number of patients admitted to ICU with intubation status between the two groups.

POD can increase morbidity and mortality, lengthen hospital stays, lead to poor functional recovery, and result in long-term decrease in cognitive function. Delirium is a complicated process, and Maldonado JR highlighted the main seven proposed mechanisms, which include neuroinflammatory, neuronal aging, oxidative stress, neurotransmitter deficiency, neuroendocrine, diurnal dysregulation, and network dysconnectivity [16]. None of these mechanisms fully explain the etiology or manifestations of delirium; rather, two or more of these lead to the biochemical derangement and, eventually, to the complex cognitive and behavioral alterations.

DEX is a kind of imidazole derivatives, which is a specific, highly selective α_2_-adrenergic receptor agonist with a receptor selectivity ratio (α_2_: α_1_) of 1620: 1. Studies have shown that DEX is beneficial in the prevention of POD for adult cardiac and non-cardiac surgical patients, administered in postoperative period [17]. According to Qian's findings [18], the traumatic stress response to splenectomy may increase the expression of IL-1β, TNF-α, Bax and caspase-3 in the hippocampus of mice, resulting in hippocampal inflammation and neuronal apoptosis, which is associated with postoperative cognitive function. DEX, on the other hand, may reverse these changes and provide protection. The administration of DEX postoperatively in elderly patients may be beneficial in preventing the development of POD. A Peking University randomized, double-blind, placebo-controlled clinical trial found that prophylactic low-dose DEX infusion (0.1 ug/kg/h from ICU admission until to 8 a.m. on the first postoperative day) can significantly reduce the incidence of POD in elderly patients admitted to ICU after noncardiac surgery [11]. Deiner S [9], on the other hand, found that intraoperative DEX infusion (0.5 ug/kg/h intraoperative and up to 2 h postoperative) did not reduce POD in elderly patients undergoing major elective noncardiac surgery. The inconsistency highlights the importance of timing when administering the drug to prevent delirium, as Deiner S suspected the result might be attributable to the short-acting nature of DEX and loss of beneficial effects after ceasing the infusion. DEX administered via PCIA is more convenient and cost-effective for patients and nurses, and it is also recommended for ward patients. A meta-analysis also pointed out that patients who received an infusion rate lower than 0.2 μg/kg/h had a significant relative risk reduction of 62% in POD, when the infusion rate was higher than 0.2 μg/kg/h had a significant 34% relative risk reduction in POD [8]. Our trial differed somewhat from theirs in that the maintenance dosage of DEX was lower in ours, but the infusion period was longer, lasting three days from the end of surgery to three days after surgery. It was important to note that the first 3 days after surgery coincided with a high incidence of postoperative delirium. Second, we observed that DEX had no effect on clinical outcomes (such as hospital length of stay, hospital non-delirium complications, or mortality). The result was controversial, and several scholars discovered the same conclusion as we did [12, 17].

In our study, no increase in the incidence of adverse events was observed in the DEX group, might be due to the small doses used, whereas the incidence of hypertension was significantly reduced in patients who received DEX. There was no difference between the two groups in postoperative cardiovascular problems, indicating that this amount is safe. In our study, DEX decreased the occurrence of PONV substantially. According to a meta-analysis [19], opioid-DEX combination analgesic techniques reduced opioid-related adverse effects such as postoperative nausea, vomiting, and pruritus when compared to opioid-only PCIA procedures.

The study has the following limitations. For starters, we did not gather information on the intensity and duration of delirium. Second, owing to the fluctuating nature of delirium, it may occur outside of the time frame we evaluate, resulting in insufficient data collection. However, our evaluators reviewed the nurse's record to see whether any patients had delirium during this period. Third, this study was a single-center study. The results may be biased due to constraints in disease type, sample size and other variables, and additional investigation is required.

## Conclusions

In conclusion, this study suggested that DEX administered via PCIA might help reduce the occurrence of delirium in elderly patients after major thoracoabdominal surgery.

### Supplementary Information

Below is the link to the electronic supplementary material.

Supplementary file1 (XLSX 63 kb)

## Data Availability

Data will be made available on request.
